# Pharmacy and nursing students’ attitudes toward nurse-pharmacist collaboration at a Chinese university

**DOI:** 10.1186/s12909-018-1285-0

**Published:** 2018-08-02

**Authors:** Shu-ping Wang, Jun Wang, Qiu-hong Huang, Ying-hong Zhang, Juan Liu

**Affiliations:** 1 Department of Pharmacy, College of Medicine, Wuhan University of Science and Technology, Wuhan, 430065 China; 2 Department of Nursing, College of Medicine, Wuhan University of Science and Technology, Wuhan, 430065 China

**Keywords:** Interprofessional relations, Nurse, Pharmacist, Attitude

## Abstract

**Background:**

The collaborative working relationship of nurses with pharmacists has increasingly captured considerable attention. This study measured pharmacy and nursing students’ attitudes toward nurse-pharmacist collaboration at a university in China.

**Methods:**

This cross-sectional study was conducted to assess the attitudes toward nurse-pharmacist collaboration using a self-developed scale delivered to a sample involving 202 nursing students and 258 pharmacy students enrolled in Wuhan University of Science and Technology.

**Results:**

Completed instruments were returned by 192 nursing students (95.0% effective response rate) and 249 pharmacy students (96.5% effective response rate). The average students’ score of attitudes toward nurse-pharmacist collaboration was 78.85 out of a total of 100. No significance was found for the attitudes toward nurse-pharmacist collaboration between two professions or between gender. The college freshmen (first-year) students had the maximum scores suggesting the most positive attitude toward nurse-pharmacist collaboration, followed by second- and third-year students, while final-year (fourth-year) students had the least.

**Conclusion:**

The students had somewhat positive attitudes toward nurse-pharmacist collaboration, but there is still room for improvement.

## Background

For the provision of patient-centered, high-quality and responsive healthcare, interprofessional team-based practice has been greatly encouraged, promoted and implemented [1]. Interprofessional team-based practice is a coordinated model involving different healthcare professionals working in a collaborative partnership with their own expertise, integrating their services with other professionals and sharing responsibility for the patients, in order to provide comprehensive and qualified healthcare according to the patient’s individual needs [2, 3]. Teamwork by the healthcare professionals has been demonstrated to provide an extensive and improved care to the patients, enhance patient safety, achieve better therapeutic outcomes, and reduce the workload of healthcare professionals [4, 5]. Recently, a great interest has been devoted to promoting collaborative professional interactions among physicians, nurses and pharmacists [6, 7]. Previously, the study focus was mainly on the contract relationship between physicians and pharmacists, or between physicians and nurses [3, 5], which could be due to the traditional leading role of physicians within the interprofessional team. However, as the professional statuses of nurses and pharmacists in interprofessional team-based practice have been continually enhanced, the collaborative working relationship of nurses with pharmacists has increasingly captured considerable attention [3, 5].

Currently, the clinical benefits associated with nurse-pharmacist collaboration have been evidenced by many studies [2, 8–15]. The nurse practitioner and pharmacist consultations in family practice resulted in large improvements in the appropriate use of medications [8]. In an Australian study [9], collaboration between community pharmacy staff and a nurse practitioner was effective in managing mental health patients with metabolic risks. In this novel service paradigm, the nurse practitioner took responsibility for the majority of patient documentation, management and referrals. No time restrictions on the nurse practitioner were imposed to complete consultations, which facilitated high-quality patient-centered care. While the pharmacists’ professional functions in medication management complemented the role of nurse practitioner. The communication of nurses and pharmacists on the basis of collaboration has been demonstrated to be efficient and cost-effective in allowing many medication discrepancies to be reconciled before causing harm, thus can prevent potential adverse drug events and improve medication safety [10, 11]. In addition, a collaborative, safety-focused nurse-pharmacist intervention to collect a medication history had been found to produce a measurable reduction of medication errors and an improvement in the accuracy of admission medication list [12]. A combined nurse/pharmacist-led clinic in primary care can improve the management of chronic pain, reduce the use of secondary care resources and achieve high rates of satisfaction [13]. In a team-based interprofessional intervention helpful for effective blood pressure control of hypertensive outpatients [2], nurses (by providing lifestyle counseling and health education) and pharmacists (by supporting patients in medication intake) worked in collaboration with physicians. Based on the regular visits for patients, nurses and pharmacists provided recommendations on medication adherence, lifestyle, and changes in therapy to physicians, who further adjusted antihypertensive therapy. Besides, Schellack et al. [14] reported that the collaborative integration of nurses and pharmacists into antimicrobial stewardship programs was effective to improve patient outcomes and reduce antimicrobial resistance. The co-operative partnerships between pharmacists and nurses ensure that the nurses are aware of the medication plan and could facilitate the early administration of time-critical medications [15].

Accordingly, interprofessional education (IPE) has been designed in nursing and pharmacy colleges/schools to improve the attitude and perception of students toward nurse-pharmacist collaboration, in order to prepare these future nurses and pharmacists for functioning in interprofessional health care teams in their future careers [16, 17]. It is well-accepted that IPE should be incorporated into health professional training programs to promote and sustain the principles of teamwork, which has been strongly encouraged by the World Health Organization [18]. Wilbur and Kelly [18] conducted the semi-structured interviews and focus groups with undergraduate pharmacy and nursing students, found the respondent students were tended to closely follow the traditional scripts (i.e. pharmacists as knowledgeable dispensers of drugs; nurses as proximate, caring aids to physicians). Such stereotypes on the each others’ roles would adversely impact the students’ future teamwork, thus reinforce the necessity and importance of IPE. It has been found that IPE interventions such as shared learning between nursing and pharmacy students could result in enhancing their professional relationship [19]. As the future pharmacy and nursing professionals, pharmacy and nursing students should have adequate awareness and receptive attitudes concerning nurse-pharmacist collaboration. According to the theory of planned behavior [20], for students, positive attitudes are prerequisite to the acceptance and subsequent behavioral change, thus could increase the effectiveness of their future interprofessional team-based clinical practice. However, no known study has investigated the students’ attitudes toward nurse-pharmacist collaboration. The present study was carried out among pharmacy and nursing undergraduates at a Chinese university to assess their attitudes toward nurse-pharmacist collaboration. The results can provide a reference for educators to implement the related curricular reforms and develop educational guidelines.

## Methods

Because there are no previous materials as references for testing attitudes toward nurse-pharmacist collaboration, an attitude scale was self-developed for this study. Items of the initial version of the instrument were either developed based on information from the relevant literatures about nurse-pharmacist collaboration or revised from the Scale of Attitudes Toward Physician-Pharmacist Collaboration, a widely-used psychometrically supported instrument to measure attitudes toward physician-pharmacist collaborative relationship [21–23]. We conducted a comprehensive review of the literatures associated with nurse-pharmacist collaboration [2, 3, 5, 8–15], which were retrieved using keywords such as “interprofessional,” “nurses,” “pharmacists,” “collaboration,” and “teamwork.” Meanwhile, 16 items from the Scale of Attitudes Toward Physician-Pharmacist Collaboration were adapted for inclusion in the initial version of the instrument.

Ten faculty with experience in the interprofessional practice or education were asked to appraise the content validity, clarity, conciseness, and relevance of the statement items included in the instrument. To assess the validity and reliability of the instrument, pretesting of the developed scale was done on a convenient sample including 10 pharmacy students and 10 nursing students. Results of the pretest were not included in final analysis of the data. The Cronbach’s alpha value was obtained as 0.80. A final instrument was prepared based on pretest results, as well as the comments and suggestions of 10 faculty and 20 students mentioned above. Thus, a final list of 25 Likert-type statement items on a 4-point scale (strongly agree = 4, agree = 3; disagree = 2; strongly disagree = 1) was included in the new instrument, named as the scale of attitudes toward nurse-pharmacist collaboration.

The final instrument was distributed to a total of 460 undergraduate students, including 202 nursing students and 258 pharmacy students, enrolled in Wuhan University of Science and Technology, a Chinese university offering a 4-year undergraduate nursing or pharmacy program. One week before the survey, faculty committee convened the mobilization meetings involving all the studied students. Then a bilingual version of the scale in Chinese and English was used to ensure the accurate comprehension of question items. The hard copies of the instrument were distributed by grade representatives and collected after completion. Participation of students was voluntary; and the respondents’ anonymity was allowed. The students were informed about the objectives of the survey in an explanatory letter on the first page of instrument. Questions were also solicited about the students’ demographic information, including profession, gender, and academic year. Estimated time to complete the instrument was approximately 30 min. Only fully completed instruments were underwent further analysis. This study was conducted for over a period of two months from March to May, 2017.

Data from instruments were coded, entered into SPSS15.0, and analysed according to the previous studies [21–23]. The internal consistency reliability of the instrument was evaluated by calculating the Cronbach’s alpha coefficient, and the corrected item-total score correlations. The underlying factor structure of the instrument was determined by principal component factor analysis with varimax rotation. The variance analysis was used to compare the differences in profession, gender, or academic year levels. Differences were considered to be significant if the *p* value was less than 0.05.

## Results

Completed instruments were returned by 192 of 202 nursing students (95.0% effective response rate) and 249 of 258 pharmacy students (96.5% effective response rate). Descriptive statistics for the students’ scores on the scale of attitudes toward nurse-pharmacist collaboration are reported in Table 1. For the 25-item final instrument, the overall Cronbach’s alpha coefficient was calculated as 0.87 in studied sample, revealing the internal consistency of this survey was reliable. For each item of the instrument, Cronbach’s alpha was calculated when the item was removed from the scale. Of the 25 items, none was found to influence the reliability of the instrument if removed. The score distribution showed a bell-shape around its mean.

**Table 1:** Descriptive Statistics for the scale of attitudes toward nurse-pharmacist collaboration administered to 441 Chinese undergraduates

Statistics	Total (*n* = 441)	Nursing students (*n* = 192)	Pharmacy students (*n* = 249)
Score, mean ± SD	78.85 ± 6.54	78.56 ± 6.62	79.07 ± 6.49
25th percentile score	74.00	74.00	75.00
50th percentile (median) score	78.00	77.00	78.00
75th percentile score	83.00	82.00	83.00
Possible score range	25—100		
Actual score range	57—99	57—97	58—99

The mean scores for nursing and pharmacy students were 78.56 ± 6.62 and 79.07 ± 6.49, respectively, out of a total of 100. There was no significant score difference found between the two professions (*p* > 0.05). As shown in Table 2, the mean scores for each item on the scale of attitudes toward nurse-pharmacist collaboration ranged from a low of 1.83 (for the reverse-score item “*A physician should be dominant authority in all healthcare matters, both nurse and pharmacist are his/her assistants*”) to a high of 3.39 (for the item “*Nurses should clarify a prescription passing the pharmacy clarification, when they feel that it might have detrimental effects on the patient*”). The corrected item-total score correlations ranged from a low of 0.06 to a high of 0.66 with a median of 0.54. The highest item-total score correlation (0.66) was obtained for the item “*Clinical discussion between the nurses and pharmacists would ensure that the nurse was aware of the medication plan and would facilitate early administration of time-critical medications*.” The lowest item-total score correlation was obtained for the item “*A physician should be dominant authority in all healthcare matters, both nurse and pharmacist are his/her assistants.*”

**Table 2:** Results of scores and factor analysis of the scale of attitudes toward nurse-pharmacist collaboration administered to 441 Chinese undergraduates

Item^a^	Scores (Mean ± SD)	Corrected item-total score correlation	Rotated Factor Coefficients^b^			
			Factor 1	Factor 2	Factor 3	Factor 4
20 Both nurses and pharmacists should contribute to offer patient education and counselling about drugs.	3.20 ± 0.51	0.55	0.67	−0.02	0.18	0.22
11 There are many overlapping areas of responsibility between nurses and pharmacists in drug treatment of the patients.	3.17 ± 0.54	0.59	0.63	0.22	0.17	0.08
19 Interprofessional relationships between nurses and pharmacists should be included in their professional education programs.	3.12 ± 0.52	0.57	0.57	0.02	0.04	0.25
21 During their education, nursing and pharmacy students should be involved in teamwork in order to understand their respective roles.	3.22 ± 0.45	0.63	0.55	0.23	0.27	0.22
9 Nurses should clarify a prescription passing the pharmacy clarification, when they feel that it might have detrimental effects on the patient.	3.39 ± 0.53	0.46	0.53	0.30	0.25	−0.30
12 Both nurses and pharmacists should be involved in optimization of the prescriptions through recommendations.	3.19 ± 0.50	0.54	0.53	0.18	0.19	0.10
13 Pharmacists should assume responsibility for improve nurses’ clinical training in drug related problems.	3.08 ± 0.53	0.56	0.45	−0.02	0.23	0.47
10 Nurses and pharmacists should be educated to establish collaborative relationships.	3.35 ± 0.46	0.64	0.44	0.46	0.11	0.25
4 Nurses can contribute to monitor adverse drug reactions and reduce the medication errors.	3.33 ± 0.51	0.57	0.40	0.46	0.35	−0.10
22 Pharmacists as well as nurses should have responsibility for monitoring the effects of drug on the patients.	3.22 ± 0.45	0.59	0.37	0.22	0.60	−0.10
6 A physician should be dominant authority in all healthcare matters, both nurse and pharmacist are his/her assistants.	1.83 ± 0.55	0.06	0.35	0.14	−0.28	0.07
2 Pharmacists are responsible for collaborating with nurses to solve drug related problems of patients in nursing practice.	3.30 ± 0.47	0.46	0.07	0.77	−0.05	0.11
1 One of most important responsibilities of the nurse is the administration of medications to patients. During this procedure, the nurse should be in collaboration with the pharmacist who provides the drug.	3.26 ± 0.47	0.51	0.14	0.62	0.05	0.18
3 Nurses can give valuable information to pharmacists regarding patient condition and medication history, which would aid a pharmacist in optimizing therapeutic plan as per patient needs.	3.35 ± 0.50	0.45	−0.02	0.59	0.28	0.05
16 Nurses play an important role in developing a collaborative relationship with pharmacist as a nurse is the most communicating person with the patient.	3.25 ± 0.52	0.54	0.18	0.45	0.27	0.18
18 Clinical discussion between the nurses and pharmacists would ensure that the nurse was aware of the medication plan and would facilitate early administration of time-critical medications.	3.24 ± 0.48	0.66	0.32	0.44	0.28	0.30
8 Prescription orders must be checked by pharmacists before nurses are carried out.	3.29 ± 0.54	0.44	0.21	0.42	0.01	0.16
17 Pharmacists should participate actively in the collaboration with nurse as a pharmacist assumes the main responsibility for pharmacotherapy outcomes.	3.09 ± 0.50	0.50	0.08	0.38	0.12	0.49
25 Imagine yourself in a situation where you work at a hospital as a nurse / pharmacist, what do you then think about the following statement: I am willing to in-corporate the pharmacotherapy for the patient with consultation of the pharmacist/nurse.	3.11 ± 0.50	0.46	0.06	0.08	0.65	0.16
24 Nurses as well as pharmacists should be involved in making policy decisions concerning the hospital/pharmacy services upon which their work depends.	3.16 ± 0.50	0.56	0.17	0.12	0.64	0.26
23 A pharmacist should be viewed as a collaborator and colleague with a nurse.	3.24 ± 0.49	0.56	0.31	0.12	0.53	0.22
7 Nurses have special expertise in patient education and medicine administration (for example, giving an injection) to patients during drug treatment.	3.00 ± 0.52	0.50	0.10	0.08	0.48	0.07
15 Nurses should be made aware that pharmacists can help in providing the right drug treatment.	3.21 ± 0.45	0.53	0.23	0.20	0.09	0.65
5 Pharmacists are a reliable source of drug information.	2.99 ± 0.56	0.42	−0.07	0.14	0.22	0.63
14 Nurse should consult pharmacists for helping patients with adverse reaction or refractory to drug treatment.	3.25 ± 0.48	0.51	0.19	0.28	0.11	0.49
Eigenvalues			6.91	1.51	1.47	1.27
Percentage of variance			27.7	6.1	5.9	5.1

Notes:

^a^Items are listed by the order of factor coefficients within each factor. Values greater than 0.35 are shown in bold. Numbers in superscript represent the sequence of the items in the actual scale. Item 6 is reverse scored.

^b^Factor 1 is considered as a construct involving “‘Interprofessional Team-based Practice.” Factor 2 is considered as a construct involving “Roles/Responsibilities for Collaborative Practice.” Factor 3 is considered as a construct involving “Relationship between Nurses and Pharmacists.” Factor 4 is considered as a construct involving “Nurses’ Experience of Pharmacist’s Role in Drug Treatment.”

Prior to factor extraction to explore the underlying construct of the scale, the Kaiser-Meyer-Olkin measure of overall sampling adequacy was calculated as 0.88, confirming that factor analysis was appropriate. The Bartlett’s test of sphericity was significant (χ^2^_(190)_ 1932.387, *p* < 0.0001), indicating that the intercorrelation matrix was factorable, thus suitable for a factor analysis. Four factors emerged with each eigenvalue greater than one (6.91, 1.51, 1.47, 1.27, respectively). Summary results of factor analysis of data for the 25 items of the scale of attitudes toward nurse-pharmacist collaboration are shown in Table 2. The first extracted factor accounting for 27.7% of the explained variance. Based on the content of 11 items with high coefficients, factor 1 can be entitled as a construct involving “Interprofessional Team-based Practice.” Factor 2 as a construct involving “Roles/Responsibilities for Collaborative Practice,” which accounted for 6.1% of the variance, consisted of 9 items with factor coefficients greater than 0.35. Factor 3 and factor 4 were considered as a construct involving “Relationship between Nurses and Pharmacists” and “Nurses’ Experience of Pharmacist’s role in Drug Treatment,” respectively.

The mean scores of attitudes toward nurse-pharmacist collaboration among students classified according to their demographic characteristics are shown in Table 3. No significant gender difference was found in scores, but the association between mean scores and the academic year levels of students was found to be statistically significant among pharmacy students (*p* < 0.01). The college freshmen (first-year) students had the maximum scores suggesting the most positive attitude toward nurse-pharmacist collaboration, followed by second- and third-year students, while final-year (fourth-year) students had the least.

**Table 3:** Group differences on the scale of attitudes toward nurse-pharmacist collaboration administered to 441 Chinese undergraduates

Group	Samples								
	Total			Nursing students			Pharmacy students		
	*n*	Score, mean ± SD	*p*	*n*	Score, Mean ± SD	*p*	*n*	Score, Mean ± SD	*p*
Gender									
Male	109	78.98 ± 6.53	0.800	11	79.18 ± 6.48	0.749	98	78.96 ± 6.85	0.831
Female	332	78.80 ± 6.42		181	78.52 ± 6.65		151	79.14 ± 6.27	
Academic year									
1st year	100	81.31 ± 5.54	< 0.001	44	79.95 ± 5.16	0.087	56	82.38 ± 5.80	< 0.001
2nd year	115	78.23 ± 6.03		51	77.67 ± 5.03		64	78.67 ± 6.94	
3rd year	113	77.71 ± 5.96		47	77.38 ± 6.18		66	77.94 ± 5.49	
4th year	108	77.21 ± 7.66		48	77.31 ± 9.12		60	77.13 ± 5.94	

## Discussion

To the best of our knowledge, this is the first study that evaluates nursing and pharmacy students’ attitudes toward nurse-pharmacist collaboration. Their average score of attitudes toward nurse-pharmacist collaboration was 78.85 out of a total of 100, suggesting the students had somewhat positive attitudes toward nurse-pharmacist collaboration. This finding is encouraging and can result in active participation of future nurses and pharmacists in nurse-pharmacist collaboration, if positive attitudes are translated into behavior. But there is still room for improvement.

Up until now, no instrument/scale has been developed specifically for measuring interprofessional attitudes and perceptions toward nurse-pharmacist collaboration. But it is worth referring to validated instruments available for measuring collaborative relationships between physicians and pharmacists. In this study, the Scale of Attitudes Toward Physician-Pharmacist Collaboration, a widely-used psychometrically supported instrument to measure attitudes toward physician-pharmacist collaborative relationships in both physicians and pharmacists, and in students of these professions [21–23], was borrowed and adapted from. Here, we developed a 25-item scale of attitudes toward nurse-pharmacist collaboration, the overall score ranges from 25 to 100 theoretically and higher total scores indicate more positive attitudes toward nurse-pharmacist collaboration. Based on the content of items loading on each factor, four underlying factors labeled as “Interprofessional Team-based Practice”(11 items), “Roles/Responsibilities for Collaborative Practice” (9 items),“Relationship between Nurses and Pharmacists”(6 items) and “Nurses’ Experience of Pharmacist’s Role in Drug Treatment” (5 items) were extracted by Varimax rotation matrix from factor analysis. These factors explained nearly half (44.6%) of the data variance supporting content validity of the scale of attitudes toward nurse-pharmacist collaboration. The 11 items in the first factor “Interprofessional Team-based Practice” accounted for 27.7% of the variance. This factor showed the importance and the implementation ways of clinical nurse-pharmacist collaboration. Moreover, the relational and organizational factors such as team communication and coordination have been considered as the key elements of interprofessional collaboration [24]. So “Roles/Responsibilities” and “Relationship” were extracted in this study in support on the multidimensionality and construct validity of the instrument. In addition, as the third largest healthcare professional group after physicians and nurses, the pharmacists are deemed to be still far from playing their due role in the healthcare system, especially in developing countries [3, 5]. But nurse as the most available communicating person has been believed to play an important role in developing the collaborative relationship with pharmacist [3]. However, the role of pharmacists appeared not to be well appreciated among nurses in healthcare setting. In a survey on the expectations and experience of nurses regarding the role of pharmacist in the Pakistan hospitals [3], only 21% nurse participants strongly agreed that the pharmacist is a professional expert on drugs. Another similar survey [5] reported only about half of the nurses agreed that, in drug administration procedure, they can receive help from pharmacist. This negative perception of nurses towards the role of pharmacists would severely impair the collaboration between nurses and pharmacists. Therefore, “Nurses’ Experience of Pharmacist’s Role in Drug Treatment” is also a key of interprofessional nurse-pharmacist collaboration, which was included as the fourth factor in the scale of attitudes toward nurse-pharmacist collaboration.

This study was conducted in a Chinese student sample. In China, interprofessional collaboration is still a relatively new concept in clinical practice [23]. The traditional paternalistic role of physicians in terms of diagnosing patients, prescribing treatment, formal decision-making, and the ultimate medical responsibility is now the mainstream in Chinese clinic setting. Accordingly, IPE has not been paid enough attention in Chinese current healthcare educational models so far. Nevertheless, along with the transformation of the educational administrative model of health professionals towards decentralised autonomy in China, grouping of health science faculties enables the development of IPE [25]. Increasing Chinese educators are beginning to carry out some endeavors to put IPE into practice [23, 26, 27]. In the present study, we found the mean score of attitudes toward nurse-pharmacist collaboration among all 441 Chinese students was 78.85 out of a total of 100. This result is comparable to our previous survey finding that the mean score of Scale of Attitudes Toward Physician-Pharmacist Collaboration for pharmacy students was 51.44 out of a total of 64, [23] and implies that Chinese undergraduates exhibit somewhat positive attitudes about nurse-pharmacist collaboration, which might support a perceptual basis and a need for nurse-pharmacist IPE in Chinese nursing and pharmacy education. However, perhaps because the nurse-pharmacist collaboration is beginning to be paid attention to, we found some unexpected observations. For example, despite the fact that the analysis on the corrected item-total correlation showed that almost all items had correlations > 0.4, implying that these item possessed a correlation with the sum of the remaining items in the scale, an item “*A physician should be dominant authority in all healthcare matters, both nurse and pharmacist are his/her assistants*” had a low correlation (0.06). In addition, a significant difference in the mean score of attitudes toward nurse-pharmacist collaboration was found among students in different academic years. In fact, in the studied Chinese university, pharmacy and nursing students complete basic course and professional basic courses during the first 2 years, and then proceed to professional courses and practice experiences during the third and final year. However, at the present stage, IPE is not well integrated into the professional curriculum in our university. Here, we found the students in higher grades appeared to express less positive attitudes toward nurse-pharmacist collaboration, which might due to the focus of current educational programs on developing uni-professional identities, the emphasis on specialization and profession-specific education, and the near complete lack of opportunities for interprofessional exchange.

Many previous studies which compared the attitudes toward interprofessional teamwork between medical and pharmacy students/ practitioners [21], or between medical and nursing students/practitioners [28–31] concluded that pharmacy or nursing students/practitioners had more positive attitudes to interprofessional collaboration than medical students/practitioners. This difference had been explained by *the principle of least interest* [21], the main idea of which is that those who have traditionally been in the weaker position are more likely to express eagerness for building collaborative relationships with others who are higher in the power hierarchy. In this study, we compared the attitudes to interprofessional collaboration between nursing and pharmacy students. However, no significance was found between two professions. The possible reason is that, compared to physicians, both nurses and pharmacists are traditionally in the weaker position during the clinical practice; and the interprofessional collaboration between nurses and pharmacists has not been paid enough attention to so far.

Based on the current situation of IPE and the students’ perceptions of nurse-pharmacist collaboration in the studied university, here we propose that, firstly, the guidelines focusing on the need to promote nurse-pharmacist collaboration in undergraduate education should be constructed by educators, in order to better prepare students for future interprofessional healthcare practice. Moreover, the educational content of nurse-pharmacist collaboration should be organically integrated in the existing educational materials, especially in professional courses and practice experiences. For example, the clinical role of pharmacists, responsibility and authority of pharmacists to ensure the effectiveness and safety of drug administration, as well as the collaborative practice between pharmacists and nurses in drug supply functions, etc. could be instilled in the nurse students. Similarly, the importance of nurses in drug-related information giving and counselling needs to be emphasized during the pharmacy training programs. In addition, some frequently studied techniques or models of teaching interprofessional practice, such as interactive learning [32], team-based learning [33], community service learning [23], etc. could be embedded in classroom teaching or extracurricular activities of nursing and pharmacy students.

This study contained several limitations. First, our results were acquired from a single institution in one country, which might limit generalisability of the findings. Second, the psychometric properties of self-developed scale of attitudes toward nurse-pharmacist collaboration used in this study should be further critically appraised, because our sample size was not big enough. Therefore, a replication of the study with a larger sample of students from multiple institutions and different countries could strengthen our findings. In addition, a bilingual version of the scale of attitudes toward nurse-pharmacist collaboration in Chinese and English was used in this study, so the validity and reliability of this scale need to be assessed in other language contexts. Despite these limitations, our findings on the students’ attitudes toward nurse-pharmacist collaboration are generally valuable and encouraging.

## Conclusion

This first survey on nurse-pharmacist collaboration among nursing and pharmacy students suggested that students had somewhat positive attitudes toward nurse-pharmacist collaboration, but there is still room for improvement. No significance was found for the attitudes toward nurse-pharmacist collaboration between two professions and between gender. But the students in higher grades appeared to express less positive attitudes toward nurse-pharmacist collaboration.

## Abbreviations

IPE: Interprofessional Education
SD: Standard Deviation
